# Recent advances in managing axial spondyloarthritis

**DOI:** 10.12688/f1000research.22577.1

**Published:** 2020-07-13

**Authors:** Priyanka Agrawal, Pedro M. Machado

**Affiliations:** 1 Department of Rheumatology, Northwick Park Hospital, London North West University NHS Trust, London, UK; 2 Department of Rheumatology, University College London Hospitals NHS Foundation Trust, London, UK; 3 Centre for Rheumatology, Division of Medicine & Department of Neuromuscular Diseases, Institute of Neurology, University College London, London, UK

**Keywords:** Axial spondyloartrhitis, ankylosing spondylitis, treatment, management, biologics, outcomes.

## Abstract

Axial spondyloarthritis (axSpA) is a chronic inflammatory rheumatic disease that predominantly affects the axial skeleton. The advent of biologic drugs has transformed the management of patients with axSpA. However, non-steroidal anti-inflammatory drugs remain the first-line drug treatment for axSpA. The optimal management of patients with axSpA requires a combination of pharmacological and non-pharmacological treatment modalities, namely exercise and physical therapy. This review looks at novel therapeutic options in patients with axSpA. It also summarises current evidence regarding radiographic progression and treat-to-target in axSpA.

## Introduction

Axial spondyloarthritis (axSpA) belongs to a clinically heterogeneous group of inflammatory rheumatic diseases that share common genetic, histological, and clinical features. It encompasses ankylosing spondylitis (AS) (or radiographic axSpA [r-axSpA]) and non-radiographic axSpA (nr-axSpA) ^1^. Over the last couple of decades, we have witnessed remarkable advances in the pathogenesis, management and treatment of axSpA. The discovery of anti-tumour necrosis factor (anti-TNF) has revolutionised the treatment of this chronic condition. More recently, interleukin-17 (IL-17) has been discovered as an alternative therapeutic target and there is promise from small molecules such as Janus kinase (JAK) inhibitors ^2^.

In this article, we shall discuss the management of axSpA with a primary focus on the recent advances. We shall review potential new biologics on the horizon. We shall also consider the use of biosimilars, radiographic progression in axSpA and the controversy surrounding the treat-to-target (T2T) approach.

## Management of axial spondyloarthritis

AxSpA can interfere with patients’ daily activities, including schooling, work, and social life ^3, 4^. The goals of treatment are to reduce disease activity (signs and symptoms), to prevent disability and structural damage, and to maintain work productivity, health-related quality of life, and social participation ^5^. Non-steroidal anti-inflammatory drugs (NSAIDs) and physical therapy remain the mainstays of treatment of axSpA. The Assessment of Spondylarthritis International Society (ASAS) and European League Against Rheumatism (EULAR) (2016 update) recommend that patients with pain and stiffness should use an NSAID as first-line drug treatment up to the maximum dose while taking risks and benefits into account. For patients who respond well to NSAIDs, continuous use of this medication is preferred in case on-demand use results in worsening of symptoms ^6^. The 2019 American College of Rheumatology/Spondylitis Association of America/Spondyloarthritis Research and Treatment Network (ACR/SAA/SPARTAN) recommendations similarly advise that adults with active axSpA receive continuous NSAIDs over on-demand NSAIDs. However, in adults with stable axSpA, on-demand treatment with NSAIDs is recommended over continuous treatment ^5^.

There is controversy regarding the role of NSAIDs in preventing radiographic progression in axSpA. Slower radiographic progression was observed in AS patients taking celecoxib continuously (that is, daily) for a 2-year period, compared with patients taking it on demand, in a study by Wanders *et al*. ^7^. A post-hoc analysis of this study showed that this effect was more pronounced in patients with elevated acute-phase reactants or in patients with a high or very high Ankylosing Spondylitis Disease Activity Score (ASDAS), which includes C-reactive protein (CRP) as one of the variables ^8^. However, this inhibitory effect on new bone formation in the spine of patients with AS was not observed in a more recent randomised multicentre trial (ENRADAS) comparing continuous diclofenac intake over the course of 2 years versus on-demand treatment ^9^. Conversely, a 2-year observational study in patients with AS showed a reduction in the progression of structural damage of the spine of patients with a high NSAID intake compared with those with a low NSAID intake. This protective effect was seen nearly exclusively in patients with syndesmophytes and elevated CRP at baseline ^10^. More recently, it was suggested that continuous use of NSAIDs reduces radiographic progression in sacroiliac joints in patients with early axSpA ^11^.

Non-pharmacological treatment modalities are important in the management of patients with axSpA. ASAS-EULAR recommend that patients should be educated about axSpA and encouraged to exercise on a regular basis and stop smoking; physical therapy should be considered ^6^. The inclusion of aerobic components, cardiorespiratory exercises, and educational programs in traditional programs of exercises may lead to improved clinical outcomes, although the most effective exercise protocol remains unclear ^12^. Promising effects of cardiorespiratory and strength exercises on emotional distress, fatigue, and ability to do a full day’s activities were shown in a small pilot Scandinavian study in patients with axSpA ^13^.

## Biologics in the treatment of axial spondyloarthritis

Until very recently there were five licensed anti-TNF drugs (adalimumab, certolizumab, etanercept, golimumab and infliximab) for the indication of AS and four (adalimumab, etanercept, certolizumab and golimumab) for the indication of nr-axSpA (in the US, only certolizumab was approved for the indication of nr-axSpA). The IL-17 blocker secukinumab has been approved by both the European Medicines Agency (EMA) and the US Food and Drug Administration (FDA) for the indication of AS. Ixekizumab was initially (2019) approved by the FDA for AS, and in July 2020, the FDA expanded the approval of ixekizumab to include nr-axSpA; almost simultaneously, the EMA also approved ixekizumab for the treatment of both AS and nr-axSpA.

ASAS-EULAR-recommended disease activity cutoffs to start anti-TNF treatment are either a Bath Ankylosing Spondylitis Disease Activity Index (BASDAI) score of at least 4 or an ASDAS of at least 2.1 after treatment with two different NSAIDs for at least 4 weeks in total ^6^. British Society of Rheumatology (BSR) and British Health Professionals in Rheumatology recommendations ^14^ define high disease activity as BASDAI and spinal pain visual analogue scale (VAS) scores of at least 4. According to the BSR, BASDAI and VAS scores should be measured on two occasions at least 4 weeks apart and patients need to have failed two NSAIDs for at least 2 weeks each unless contraindicated.

The 2019 ACR-SAA-SPARTAN recommendations also advise anti-TNF drugs in patients with axSpA when activity persists despite NSAID treatment. Anti-TNF monoclonal antibodies should be preferred in patients with concomitant inflammatory bowel disease (IBD) or recurrent iritis. According to these recommendations, IL-17 blockers are advised for patients with active disease who have heart failure or demyelinating disease as a contraindication to anti-TNF and in primary non-responders to anti-TNF. Secukinumab and ixekizumab are not recommended in patients with IBD or recurrent uveitis. In the 2019 ACR-SAA-SPARTAN recommendations, tofacitinib, a JAK inhibitor currently not approved for axSpA, is highlighted as a potential second-line option for patients with contraindications to a TNF inhibitor (TNFi) other than infections. Recommendations regarding tofacitinib may change pending the results of larger clinical trials ^5^. Owing to the likelihood for symptom recurrence, discontinuation of biologics is not recommended by ACR-SAA-SPARTAN. If tapering is considered, patients should be counselled regarding the potential for increased disease activity ^5^. In Table 1, an overview of biological disease-modifying anti-rheumatic drugs (DMARDs) and targeted synthetic DMARDs in axSpa and related chronic inflammatory conditions is presented.

**Table 1.** Overview of biological disease-modifying anti-rheumatic drugs (DMARDs) and targeted synthetic DMARDs in axial spondyloarthritis and related chronic inflammatory conditions.

Target	Drug	Axial spondyloarthritis	Psoriatic arthritis	Psoriasis	Crohn’s disease	Rheumatoid arthritis	Uveitis
**TNF**	Adalimumab (mAb to TNF)						
	Certolizumab (mAb to TNF)						
	Etanercept (fusion protein against TNF)						
	Golimumab (mAb to TNF)						
	Infliximab (mAb to TNF)						
**IL-1**	Anakinra (IL-1R antagonist)						
**B cells**	Rituximab (mAb to CD20)						
**T cells**	Abatacept (inhibitor of T-cell co- stimulation)						
**IL-6**	Tocilizumab (mAb to IL-6R)						
	Sarilumab (mAb to IL-6R)						
**IL-17**	Secukinumab (mAb to IL-17A)						
	Ixekizumab (mAb to IL-17A)						
	Brodalumab (mAb to IL-17R)						
	Bimekizumab (mAb to IL-17A and IL-17F)						
	Netakimab/BCD-085 (mAb to IL-17R)						
**IL-12 and** **IL-23**	Ustekinumab (mAb to IL-12/23p40)						
	Guselkumab (mAb to IL-23p19)						
	Tildrakizumab (mAb to IL23p19)						
	Risankizumab (mAb to IL23p19)						
**PDE4**	Apremilast (PDE4 inhibitor)						
**JAK**	Tofacitinib (JAK1/3 inhibitor)						
	Filgotinib (JAK1 inhibitor)						
	Upadacitinib (JAK1 inhibitor)						
**GM-CSF**	Namilumab (mAB to GM-CSF)						

Key:	
	Efficacy shown in at least one randomised controlled trial
	Some data from pilot or proof-of-concept trials suggest a positive effect
	Some data from pilot or proof-of-concept trials suggest a lack of effect
	Lack of efficacy shown in at least one randomised controlled trial
	No conclusive data available about efficacy

GM-CSF, granulocyte macrophage-colony-stimulating factor; IL, interleukin; JAK, Janus kinase; mAb, monoclonal antibody; PDE4, phosphodiesterase 4; TNF, tumour necrosis factor

## Biologic drugs that have recently shown efficacy

The efficacy of ixekizumab, a high-affinity monoclonal antibody (mAb) against IL-17A, has been demonstrated in both biologic-naïve and TNFi-experienced patients with r-axSpA (NCT02696785 and NCT02696798) ^15– 17^. More recently, the first study demonstrating the efficacy of ixekizumab in nr-axSpA was also published ^18^. Positive secukinumab (NCT02696031) data in nr-axSpA were also recently presented in abstract form, but the manuscript has yet to be published ^19^.

Bimekizumab, a mAb that potently and selectively neutralises both IL-17A and IL-17F, has demonstrated substantial improvements in both musculoskeletal and skin outcomes ^20^. Brodalumab, a human anti-IL-17 receptor A mAb has also demonstrated efficacy in phase 3 studies in both AS and nr-axSpA ^21^. The bimekizumab and brodalumab studies have been published in abstract form only.

Netakimab/BCD-085, a humanised mAb against IL-17 with genetically modified Fc- and CDR-regions, has shown efficacy in active AS in a dose-finding phase 2 clinical trial (abstract publication ^22^).

JAK inhibitors are emerging as an effective therapeutic approach in patients with axSpA. Both tofacitinib (JAK1–3 inhibitor) and filgotinib (selective JAK1 inhibitor) have shown efficacy and safety in phase 2 placebo-controlled trials of patients with AS. Tofacitinib was superior to placebo in reducing signs, symptoms and objective endpoints of patients with active AS and had a safety profile similar to what has been reported in the literature ^23^. More recently, van der Heijde *et al*. showed that filgotinib was efficacious and safe for patients with active AS and inadequate response or intolerance to NSAIDs ^24^. Therefore, further investigation of filgotinib in AS is warranted ^24^ (NCT03117270). Finally, results of the first randomised trial of upadacitinib (selective JAK1 inhibitor) versus placebo in patients with AS were recently published; upadacitinib was efficacious and well tolerated in patients with active AS who had not responded to or had a contraindication to treatment with NSAIDs. These data support further investigation of upadacitinib for the treatment of axSpA ^25^.

## Biologic drugs that have not shown efficacy

Uncontrolled or controlled trials with anakinra (IL-1 blocker) ^26, 27^, abatacept (inhibitor of T-cell co-stimulation) ^28^, rituximab (B-cell depletion agent) ^29, 30^ and tocilizumab (IL-6 blocker) ^31, 32^ have shown no consistent efficacy in patients with AS. Of note, conventional synthetic DMARDs have also failed to show efficacy in axial disease.

Apremilast is a small-molecule inhibitor of phosphodiesterase 4 (PDE4) that modulates the inflammatory response. In a small (n = 38), 12-week, double-blind, placebo-controlled, phase 2 study of symptomatic AS patients with positive magnetic resonance imaging (MRI) ^33^, apremilast did not meet its primary endpoint (change in BASDAI score at week 12) but was associated with numerically greater improvement in all clinical assessments compared with placebo. A subsequent double-blind, placebo-controlled, phase 3 study assessing the efficacy and safety of apremilast in active AS was completed (NCT01583374). Preliminary online reports suggest that this was a negative study (primary endpoint not met: ASAS20 at week 16) (source: NCT01583374). This study has not been published yet.

In a randomised, double-blind, placebo-controlled, dose-finding phase 2 study, treatment with risankizumab (a humanised mAb targeting IL-23A) did not show evidence of clinically meaningful improvements compared with placebo in patients with active AS; the primary endpoint, ASAS40 response at week 12, was not met. This study suggested that despite a genetic association with the IL-23 pathway, IL-23 may not be a relevant driver of disease pathogenesis and clinical manifestations in AS (NCT02047110) ^34^.

In three placebo-controlled trials, the efficacy of ustekinumab (an anti-IL-12/23 antibody) in the treatment of axial SpA was not demonstrated. The safety profile was consistent with that of studies in other indications ^35^ (NCT02437162, NCT02438787 and NCT02407223).

Siebert *et al*. recently published an editorial on the reasons for failure of IL-23p19 inhibition in AS ^36^. The authors hypothesise that established AS may have “transitioned pathogenetically” to a mature type 17 phenotype, making it unresponsive to IL-23p19 blockade and other upstream treatment strategies, such as IL-6 inhibition, that would normally modulate the IL-17 response ^36^. Therefore, neutralising upstream molecules would be less effective than specifically targeting IL-17A. Furthermore, other effector pathologic pathways driving the IL-17 response are probably still unknown and need to be identified ^36^. In Table 2, ongoing axSpA drug trials are presented.

**Table 2.** Ongoing clinical trials in axial spondyloarthritis.

Drug	Mechanism of action	Study design	Patient group	Trial number
Secukinumab (intravenous)	Human monoclonal IgG1 kappa antibody against IL-17	Phase 3, multicentre, DB RCT	axSpA	NCT04156620
Ixekizumab	Humanised mAb against IL-17A	Long Term Extension Study of a Phase 3, multicentre, DB RCT	axSpA	NCT03129100
Brodalumab	Human mAb that binds to the IL-17 receptor	Phase 3, multicentre, DB RCT	axSpA	NCT02985983
Bimekizumab	Humanised monoclonal IgG1 antibody against IL-7A and IL-17F	Phase 3, multicentre, DB RCT	r-axSpA nr-axSpA	NCT03928743 NCT03928704
Tildrakizumab	Human mAb against IL-23	Phase 3, multicentre, open label, non-randomised	axSpA	NCT03552276
Netakimab/BCD-085	Humanised mAb against IL-17 with genetically modified Fc- and CDR-regions	Phase 3, multicentre, DB RCT	r-axSpA	NCT03447704
Upadacitinib	JAK1 selective inhibitor	Phase 3, DB RCT	axSpA	NCT04169373
Namilumab	Human monoclonal IgG1 kappa antibody against GM-CSF	Phase 2a, proof-of-concept, DB RCT	axSpA	NCT03622658

axSpA, axial spondyloarthritis; DB RCT, Double-Blind Placebo-Controlled Randomised Clinical Trial; GM-CSF, granulocyte-macrophage colony-stimulating factor; IL, interleukin; mAb, monoclonal antibody; nr-axSpA, non-radiographic axial spondyloarthritis; r-axSpA, radiographic axial spondyloarthritis

## Biosimilars

The World Health Organization has defined a “similar biotherapeutic product” (also called “biosimilar”) as a biotherapeutic product that is similar in terms of quality, safety and efficacy to an already licensed original or “reference biotherapeutic product” (also called “originator” drug) ^37^. In adults with stable AS receiving an originator anti-TNF, ACR-SAA-SPARTAN strongly recommends continuing treatment with the originator anti-TNF over mandated switching to its biosimilar ^5^. The BSR published a position statement ^38^ on the use of biosimilars in clinical practice. The key principles of this document are that (1) all biologics and biosimilars should be prescribed by brand name rather than by international non-proprietary name, (2) clinical effectiveness and patient safety should be the overriding principles for prescribing any biologic agent, (3) substitution should be done only with the consent of the prescribing clinician, (4) decisions should be made in partnership with the patients, and (5) registration with the BSR Biologic Registers or other appropriate UK register is recommended. Therefore, switching should be based on a shared decision-making process between patients and rheumatologists, should be a clinically informed decision not made solely for economic reasons, and should take contextual factors of the health-care system into account ^39^.

Biosimilars are now often prescribed as the first biologic drug treatment, and many patients have been successfully switched from originator drugs to the biosimilar. They have resulted in substantial savings on the use of biologic therapy, providing greater accessibility to patients. There are now biosimilars for adalimumab (Amgevita, Cyltezo, Hyrimoz and Imraldi), etanercept (Benepali, Erelzi and Eticovo) and infliximab (Inflectra, Ixifi, Remicade, Remsima and Renflexis) and many more being produced/tested.

## Role of biologics in the inhibition of radiographic progression

The clinical efficacy of biologics in axSpA has been robustly demonstrated. However, it is still unclear whether they can prevent the progression of structural damage, particularly new bone formation. There are also methodological challenges that have contributed to this uncertainty, namely the fact that radiographic progression is a slow process that requires at least 2 years in order to be reliably detected and the ethical impossibility of performing a comparative study with a 2-year placebo arm (given the known clinical efficacy of biologics in patients who do not respond to conventional treatments) ^2^.

Importantly, radiographic progression is a heterogeneous process that varies significantly not only between patients but also within the same patient (bursts of radiographic progression can be followed by periods of a relatively low rate of progression or even no progression). Some risk factors for radiographic progression have been identified: the presence of syndesmophytes (the strongest risk factor; previous damage predicts further damage), male gender, HLA-B27 positivity, long disease/symptom duration, high CRP, MRI activity, high disease activity and smoking. It is hypothesised that more intensive T2T approaches might contribute to decreasing the rate of radiographic progression, particularly in high-risk groups ^40^.

Karmacharya *et al*. ^40^ recently undertook a systematic review and meta-analysis on the effect of different therapies on radiographic progression in axSpA and suggested a protective effect of TNFi treatment on spinal radiographic progression of AS after at least 4 years of treatment in studies with low risk of bias. Observational data therefore suggest that anti-TNF treatment might prevent new bone formation in axSpA. Regarding NSAIDs (also discussed above) and secukinumab, only data up to 2 years were available, and no effect was found.

SURPASS is an ongoing trial comparing the effect of secukinumab on radiographic progression in AS as compared with an adalimumab biosimilar ^41^. The primary endpoint of this first head-to-head, phase 3b, randomised, biologic-controlled study is “the absence of progression as measured by the modified Stoke Ankylosing Spondylitis Spine Score” ^41^. Hopefully, this study will give us more information regarding the comparative structural efficacy of TNF blockers versus IL-17 blockers, thus contributing to evidence-based decision-making.

## Treat-to-target approach

The concept of T2T strategies is well founded in the management of chronic diseases that have a well-defined and usually very objective measure that is highly associated with future health outcomes and evidence that maintaining the levels of this measure below a certain threshold will lead to better long-term health ^42^. The T2T approach in axSpA is indirectly supported by associations between levels of axSpA disease activity (particularly ASDAS) and future radiographic progression but lacks robust direct evidence. The 2019 ACR-SAA-SPARTAN recommendations mention that “focus on a specific target could lead to rapid cycling through all currently available treatments in some patients” ^5^. The 2016 update of the ASAS/EULAR recommendations for the management of axSpA recommends that “treatment should be guided according to a predefined treatment target” but controversy remains as to what this target should be ^6^. Importantly, this document also states that “the target should be a shared decision between patient and rheumatologist, taking all relevant situational factors into consideration” ^6^. However, data from a randomised controlled trial comparing the efficacy of a T2T approach versus standard of care in axSpA are still lacking. Furthermore, the cost effectiveness of a T2T strategy in clinical practice will require testing ^43^.

The 2017 international task force update of recommendations on T2T in axSpA and psoriatic arthritis ^44^ advises that the treatment target should be clinical remission/inactive disease of musculoskeletal (arthritis, dactylitis, enthesitis and axial disease) and extra-articular manifestations. In axSpA, the ASDAS is a preferred measure to define the target.

The Tight Control in Spondyloarthritis (TICOSPA) study is a prospective, randomised (cluster) study to evaluate the potential benefit of a T2T approach in comparison with standard of care in patients with axSpA (TICOSPA, NCT03043846). Results from this study were presented in June at the EULAR 2020 conference (THU0370); the authors reported that usual care resulted in a good outcome in a substantial number of patients but the tight control and treat-to-target strategy was not superior for the primary outcome (ASAS Health Index) despite a greater number of bDMARDs prescription; nevertheless, a general trend in favour of the tight control arm was observed, with a comparable safety profile, and was found to be favourable from a societal health economic perspective. A German 1-year randomised controlled study taking an intense treatment approach versus routine treatment (STRIKE, NCT02897115) was prematurely terminated because of slow recruitment. Another phase 3 clinical trial, the Treat-to-target in Axial Spondyloarthritis (AScalate) study, is currently recruiting and is a randomised, open-label multicentre trial to investigate the efficacy of a T2T treatment strategy with secukinumab as a first-line biologic compared with a standard-of-care treatment over 36 weeks in patients with active axSpA (NCT03906136). A phase 4 trial, the Treat-to-target With Secukinumab in Axial Spondyloarthritis (TRACE) study, is also looking at T2T with secukinumab in axSpA (NCT03639740).

## Conclusions

With so many new biologics on the horizon, the future looks promising for managing patients with axSpA. However, there is an unmet need for results from head-to-head studies directly comparing the efficacy and safety of different biologics in axSpA. This would help determine the optimal sequencing of treatments. There are limitations in using disease activity scores and radiographic progression as markers of treatment response and hence research into suitable biomarkers needs to be undertaken.
